# External validation of the QLifetime cardiovascular risk prediction tool: population cohort study

**DOI:** 10.1186/s12872-023-03209-8

**Published:** 2023-04-15

**Authors:** Shona Livingstone, Daniel R. Morales, Jacques Fleuriot, Peter T. Donnan, Bruce Guthrie

**Affiliations:** 1 Division of Population Health and Genomics, University of Dundee, Dundee, UK; 2 School of Informatics, University of Edinburgh, Edinburgh, UK; 3 Advanced Care Research Centre, Usher Institute, University of Edinburgh, Old Medical School, University of Edinburgh, Doorway 3, Teviot Place, Edinburgh, EH8 9AG UK

**Keywords:** Cardiovascular risk, Primary prevention, Risk prediction, Lifetime models, External validation, QLifetime, Competing mortality risk

## Abstract

**Background:**

Prediction of lifetime cardiovascular disease (CVD) risk is recommended in many clinical guidelines, but lifetime risk models are rarely externally validated. The aim of this study was to externally validate the QRiskLifetime incident CVD risk prediction tool.

**Methods:**

Independent external validation of QRiskLifetime using Clinical Practice Research Datalink data, examining discrimination and calibration in the whole population and stratified by age, and reclassification compared to QRISK3. Since lifetime CVD risk is unobservable, performance was evaluated at 10-years’ follow-up, and lifetime performance inferred in terms of performance for in the different age-groups from which lifetime predictions are derived.

**Results:**

One million, two hundreds sixty thousand and three hundreds twenty nine women and 1,223,265 men were included in the analysis. Discrimination was excellent in the whole population (Harrell’s-C = 0.844 in women, 0.808 in men), but moderate to poor stratified by age-group (Harrell’s C in people aged 30–44 0.714 for both men and women, in people aged 75–84 0.578 in women and 0.556 in men). Ten-year CVD risk was under-predicted in the whole population, and in all age-groups except women aged 45–64, with worse under-prediction in older age-groups. Compared to those at highest QRISK3 estimated 10-year risk, those with highest lifetime risk were younger (mean age: women 50.5 vs. 71.3 years; men 46.3 vs. 63.8 years) and had lower systolic blood pressure and prevalence of treated hypertension, but had more family history of premature CVD, and were more commonly minority ethnic. Over 10-years, the estimated number needed to treat (NNT) with a statin to prevent one CVD event in people with QRISK3 ≥ 10% was 34 in women and 37 in men, compared to 99 and 100 for those at highest lifetime risk.

**Conclusions:**

QRiskLifetime underpredicts 10-year CVD risk in nearly all age-groups, so is likely to also underpredict lifetime risk. Treatment based on lifetime risk has considerably lower medium-term benefit than treatment based on 10-year risk.

**Supplementary Information:**

The online version contains supplementary material available at 10.1186/s12872-023-03209-8.

## Background

Although the incidence of cardiovascular disease(CVD) has fallen in most developed countries over the last 30 years, CVD remains one of the most common causes of morbidity and mortality worldwide. Prevention of CVD is therefore a policy priority, and a key practical question is who should be targeted for pharmacological primary prevention. In relation to initiation of statins, risk prediction tools are usually recommended by guidelines for the primary prevention of CVD to target treatment at people above a specified threshold of predicted risk. Prediction tools typically predict either over a fixed time (often ten years) or over a lifetime. Lifetime risk prediction is argued to be more appropriate in younger people who may not exceed a particular 10-year risk threshold even though they have markedly unfavourable CVD risk profiles (mitigated in the short-term by being young) and are at high risk of premature CVD beyond 10-years [1–5]. Lifetime risk models also appropriately account for competing mortality risk, which is ignored and a cause of over-prediction in many CVD risk prediction tools [6–8]. Lifetime CVD risk prediction tools are recommended to guide treatment in several international guidelines, although there is no consensus on what threshold of lifetime risk should trigger an offer of statin treatment [1]. Lifetime risk prediction is not currently recommended for CVD risk stratification by the National Institute for Health and Care Excellence (NICE) [9], but NICE have identified lifetime risk prediction as a topic to examine further in a future guideline update [10]. In the UK, the QRiskLifetime prediction tool is available as a standalone web-based tool [11] or as the risk engine underlying the Joint British Societies risk calculator (JBS3) [2] and heart age [12] tools.

External validation of CVD risk prediction tools is needed before they are widely implemented, but lifetime models are difficult to validate since observational datasets do not have lifetime follow-up. The same is also true in the datasets used to derive lifetime risk prediction, including the QRiskLifetime derivation dataset [3]. In derivation, lifetime CVD risk is therefore estimated by using shorter-term observed CVD rates at different ages to infer what would happen to someone in the future, under the assumption that age-specific incidence of CVD will not change in the meantime. The same effectively applies in validation, which can only be done over shorter time-scales [3, 5], with true lifetime performance inferred by performance in different age-groups. The aim of this paper is to externally validate the QRiskLifetime CVD prediction model in a large UK primary care dataset using a 10-year time horizon, and to explore recalibration compared to QRISK3.

## Methods

The overall design of the study is an independent external validation of a risk prediction (prognosis) model, designed and reported consistent with TRIPOD guidelines [13].

### Data source and population

Analysis used Clinical Practice Research Datalink (CPRD) Gold) [14], which includes linked primary care, hospital and mortality data. Patients were eligible if they: were permanently registered with a practice contributing up-to-standard data for at least one year and with linkage to Hospital Episode Statistics (HES) discharge and Office of National Statistics (ONS) mortality data, and had no prior history of CVD or statin treatment. Cohort entry was defined as the latest of 01/01/04, a patient’s 30th birthday, or contribution of up-to-standard data for at least 1 year. Cohort exit was the earliest of: first CVD event; death; prescription of a statin; deregistration from the practice; end of data collection from the practice; or end of study on 31/3/16. All outcomes and predictors were recorded as part of routine clinical care, and therefore recorded blind to the study hypothesis. No formal power calculation was done, as the study size is determined by the data available in CPRD which was considered sufficient [15].

### Outcomes

A first CVD event was defined as the earliest recording of any fatal or non-fatal coronary heart disease (CHD), ischaemic stroke, or transient ischaemic attack, recorded as ICD-10 codes in HES admissions or as the underlying cause of death in ONS death registration data, or as Read codes in GP electronic health records. ICD-10 and Read codes defining outcomes are those used in QRISK3 derivation [16] (detailed in a previous paper [6]).

### Prediction model

We used publicly available QRISK®-lifetime-2011 software to calculate QRiskLifetime scores for each patient to age 95 and additionally constrained to a 10-year prediction horizon (under GNU Lesser General Public Licence v3). Predictor variables including body mass index, smoking, cholesterol and blood pressure were ascertained from GP electronic health records. All predictor variables are listed in Table 1. Our cohort matched the QRiskLifetime derivation sample and methods with some exceptions, namely: (1) We used a cohort entry date of 1/1/04 rather than 1/1/98; (2) When calculating baseline values, the derivation paper included cholesterol values measured *after*cohort entry, whereas we only included cholesterol values measured before cohort entry; and (3) Individual Townsend deprivation scores were not available, so we used the median of the vigintile (equal 20th ) of score that an individual lived in. Predictor codesets used and methods of data handling have been previously published [6].

**Table 1:** Baseline data compared to QRiskLifetime derivation cohort

	Women external validation cohort *N* = 1,260,329	Men external validation cohort *N* = 1,223,265	All patients QRiskLifetime internal validation cohort [3] *N* = 1,267,159
Mean (SD) Age (years)	49.3 (14.2)	47.6 (13.0)	48.0 (14.2)
Mean (SD) Body mass index	26.2 (5.8)	26.8 (4.6)	26.1 (4.5)
Median (IQR) Townsend score	-1.5 (-2.5 to 0.5)	-1.5 (-2.5 to 0.5)	-0.3 (3.5)^a^
Mean (SD) Total cholesterol:HDL cholesterol ratio	3.7 (1.1)	4.4 (1.3)	4.2 (1.3)
Mean (SD) Systolic blood pressure (mmHg)	127 (18)	132 (16)	131.7 (20.5)
Ethnicity No. (%)			
White or not recorded	1,168,417 (92.7)	1,155,055 (94.4)	1,219,987 (96.3)
Indian	16,627 (1.3)	12,346 (1.0)	7577 (0.6)
Pakistani	6546 (0.5)	5031 (0.4)	3663 (0.3)
Bangladeshi	1649 (0.1)	1604 (0.1)	2632 (0.2)
Other Asian	10,118 (0.8)	7946 (0.6)	5032 (0.4)
Black Caribbean	8154 (0.6)	5913 (0.5)	4666 (0.4)
Black African	14,495 1.2)	10,681 (0.9)	9471 (0.8)
Chinese	5135 (0.4)	2917 (0.2)	3068 (0.2)
Other	29,188 (2.3)	21,772 (1.8)	11,063 (0.8)
Smoking status No. (%)^b^			
Non-smoker	585,281 (59.3)	403,983 (48.4)	631,545 (49.8)
Former smoker	189,719 (19.2)	198,717 (23.8)	193,974 (15.3)
Light smoker	63,592 (6.4)	58,543 (7.0)	71,037 (5.6)
Moderate smoker	91,518 (9.3)	90,692 (10.9)	91,679 (7.2)
Heavy smoker	56,241 (5.7)	83,169 (10.0)	74,056 (5.8)
FH of CHD in first degree relative < 60 years	88,164 (7.0)	68,814 (5.6)	143,593 (11.3)
Type 2 diabetes	16,744 (1.3)	20,883 (1.7)	20,868 (1.7)
Treated hypertension	115,548 (9.2)	82,387 (6.7)	67,986 (5.4)
Atrial fibrillation	8164 (0.6)	10,528 (0.9)	6589 (0.5)
Chronic kidney disease	6675 (0.5)	5403 (0.4)	1917 (0.2)
Rheumatoid arthritis	12,357 (1.0)	4590 (0.4)	Not reported
Charlson score^c^			
0	996,700 (79.1)	1,005,402 (82.2)	Not reported
1	198,089 (15.7)	173,274 (14.2)	
2	50,105 (4.0)	33,558 (2.7)	
3+	15,435 (1.2)	11,031 (0.9)	

^a^Validation study reports mean (standard deviation)

^b^For this study, % of non-missing; for QRiskLifetime derivation paper % of all patients

^c^All listed variables are used as predictors in the QRiskLifetime model apart from Charlson score which is not included in the prediction model but is used as a stratifying variable in analysis of discrimination and calibration

### Missing data

Supplementary Table S1 details the extent of missing data and how missingness was handled. Multivariate Imputation by Chained Equations [17] was used to generate five imputed datasets for missing body mass index (BMI), total cholesterol:HDL cholesterol ratio (TC:HDL), systolic blood pressure (SBP), and smoking status. Analyses of these datasets were combined using Rubin’s rules [18] to give summary point estimates with confidence limits that reflect the added uncertainty associated with imputing missing values.

### Statistical methods

The lifetime (to age 95) and 10-year risk of experiencing a cardiovascular event was calculated for each patient using QRISK®-lifetime-2011 software without recalibration. The performance of the risk score was assessed by examining discrimination and calibration of the model over a 10-year time horizon, in the whole population and stratified by age-group and Charlson Comorbidity Index (CCI) at study entry [19].

Discrimination is the ability of the risk score to differentiate between patients who experience a CVD event during follow-up and patients who do not. Discrimination was evaluated using Harrell’s C-statistic (a C-statistic of 1 indicates perfect discrimination, whereas a C-statistic of 0.5 indicates discrimination no better than chance; we interpreted values > 0.8 as showing excellent discrimination, 0.6–0.79 as moderate, and 0.5–0.59 as poor), Royston and Sauerbrei’s D statistic (higher values indicate greater discrimination) and an R-squared statistic of explained variation in censored survival data [20, 21].

Calibration refers to how closely predicted risk and observed probabilities agree at group-level. This was assessed for equally-sized groups of participants ranked by predicted risk. Calibration of the risk score predictions was assessed by plotting observed proportions with an event versus predicted probabilities. Since QRiskLifetime accounts for competing mortality risk, we evaluated calibration using the Aalen-Johansen estimator of observed risk which accounts for the competing risk of non-CVD death and therefore estimates the cumulative incidence of CVD [22]. Calibration plots were generated separately by sex for all patients and for subgroups of age and modified Charlson Comorbidity Index.

Consistent with the validation of QRiskLifetime over a 10-year time horizon, we examined changes in which patients were recommended for treatment based on either QRISK3 or QRiskLifetime 10-year predicted risk of ≥ 10% (the threshold recommended by the UK National Institute for Health and Care Excellence [23]). We calculated the Net Reclassification Index (NRI) with bootstrapped 95% confidence intervals at the 10-year 10% predicted risk threshold. NRI was calculated for people experiencing a CVD event (NRI+), for people not experiencing a CVD event (NRI-) and overall (NRI). NRI examines the extent to which using QRiskLifetime is better at classifying cases who experience the event as high-risk (10-year risk ≥ 10%) and non-cases as low risk (10-year risk < 10%). Since there is no recommended threshold of lifetime risk at which to define an individual as high-risk, we also compared which patients were recommended for treatment by QRISK3 at the 10% threshold and by QRiskLifetime using a threshold defined to identify the same number of patients (i.e. if QRISK3 recommended 19.0% of patients for treatment, we selected the 19.0% of patients at highest lifetime risk). For both comparisons, we examined the characteristics of patients recommended for treatment, the observed risk of CVD at 10 years, and the number needed to treat (NNT) to prevent one new CVD event assuming all people recommended for treatment actually took a statin assuming a relative risk reduction of 25% for new CVD events. All models were fitted in R v4.1.0.

## Results

There were 1,260,329 women with mean age 49.3 (SD 14.2) years and 1,223,265 men with mean age 47.6 (SD 13.0) years in the external validation cohorts. Compared to the QRiskLifetime internal validation cohort [3], there was: a larger proportion of people from minority ethnic backgrounds; fewer people with a recorded family history of premature CVD; a higher proportion of treated hypertension; and somewhat higher proportions of atrial fibrillation and chronic kidney disease (Table 1). There were higher proportions with missing data in this study than the original study, likely reflecting the use of data recorded after cohort entry date in the derivation study (Supplementary Table 1).

Median follow-up was 5.7 (interquartile range [IQR] 2.2–10.2) years in women and 5.2 (IQR 2.0-9.3) years in men, similar to the QRISK3 cohort [16]. Crude incidence of CVD was higher in men than women (7.5 vs. 5.5 CVD events/1000 person-years), and increased markedly with age (Supplementary Table S2). Non-cardiovascular death had similar incidence to CVD in women, whereas in men incident cardiovascular disease was more common in men up to age 65–69 years, with non-cardiovascular more common subsequently (Supplementary Table S3 and Figure S1).

In the entire population over 10-years, QRiskLifetime discrimination was excellent in both women (C = 0.844 in this study vs. area under receiver operating curve [AUROC] 0.842 in original study internal validation [3]) and men (C = 0.808 vs. AUROC = 0.828 in internal validation [3]) (Table 2). However, when stratified by age, discrimination was only moderate in younger age-groups and was poor in people aged 75–84 (C = 0.578 in women, 0.556 in men). Stratified by CCI, discrimination was excellent in people with low morbidity (CCI = 0 or 1) but only moderate in people with high morbidity (in women with CCI = 3+, C = 0.724; in men with CCI = 3+, C = 0.656).

**Table 2:** Discrimination and model fit (evaluated at 10 years follow-up)

	Women			Men		
	Harrell’s C (95% CI)	D (95% CI)	R-squared (95% CI)	Harrell’s C (95% CI)	D (95% CI)	R-squared (95% CI)
All patients	0.844 (0.841,0.847)	2.19 (2.17,2.21)	53.3 (52.9,53.7)	0.808 (0.806,0.811)	1.87 (1.85,1.89)	45.5 (45.1,46.0)
Age group						
30–44	0.714 (0.703,0.725)	1.33 (1.26,1.39)	29.6 (27.6,31.7)	0.714 (0.706,0.722)	1.24 (1.20,1.29)	26.9 (25.6,28.3)
45–64	0.692 (0.687,0.698)	1.14 (1.10,1.17)	23.5 (22.5,24.6)	0.671 (0.667,0.675)	0.97 (0.94,0.99)	18.2 (17.4,19.1)
65–74	0.631 (0.625,0.637)	0.75 (0.71,0.79)	11.8 (10.6,13.0)	0.597 (0.591,0.603)	0.54 (0.51,0.58)	6.6 (5.8,7.3)
75–84	0.578 (0.573,0.583)	0.44 (0.40,0.49)	4.5 (3.6,5.5)	0.556 (0.549,0.562)	0.32 (0.28,0.36)	2.4 (1.9,3.0)
CCI						
0	0.844 (0.840,0.848)	2.19 (2.17,2.21)	53.4 (52.8,53.9)	0.803 (0.800,0.806)	1.82 (1.80,1.84)	44.1 (43.6,44.6)
1	0.820 (0.814,0.826)	1.95 (1.92,1.99)	47.6 (46.7,48.5)	0.798 (0.792,0.804)	1.76 (1.72,1.80)	42.4 (41.3,43.5)
2	0.768 (0.758,0.779)	1.54 (1.49,1.60)	36.3 (34.6,37.9)	0.701 (0.690,0.711)	1.13 (1.07,1.19)	23.4 (21.5,25.3)
3+	0.724 (0.708,0.740)	1.29 (1.21,1.38)	28.5 (25.8,31.2)	0.656 (0.639,0.673)	0.91 (0.82,0.99)	16.4 (13.8,19.1)

*CCI* Charlson Comorbidity Index

**Fig. 1:**
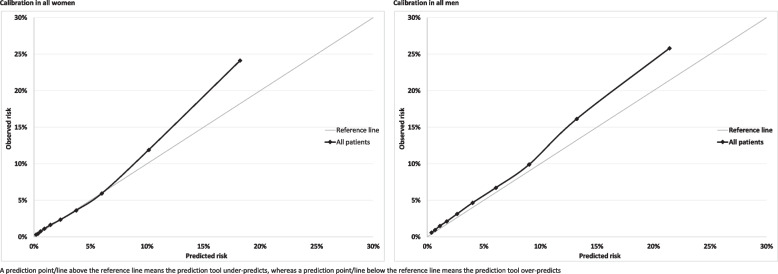
Calibration in women (left hand) and men (right hand) for whole population

**Fig. 2:**
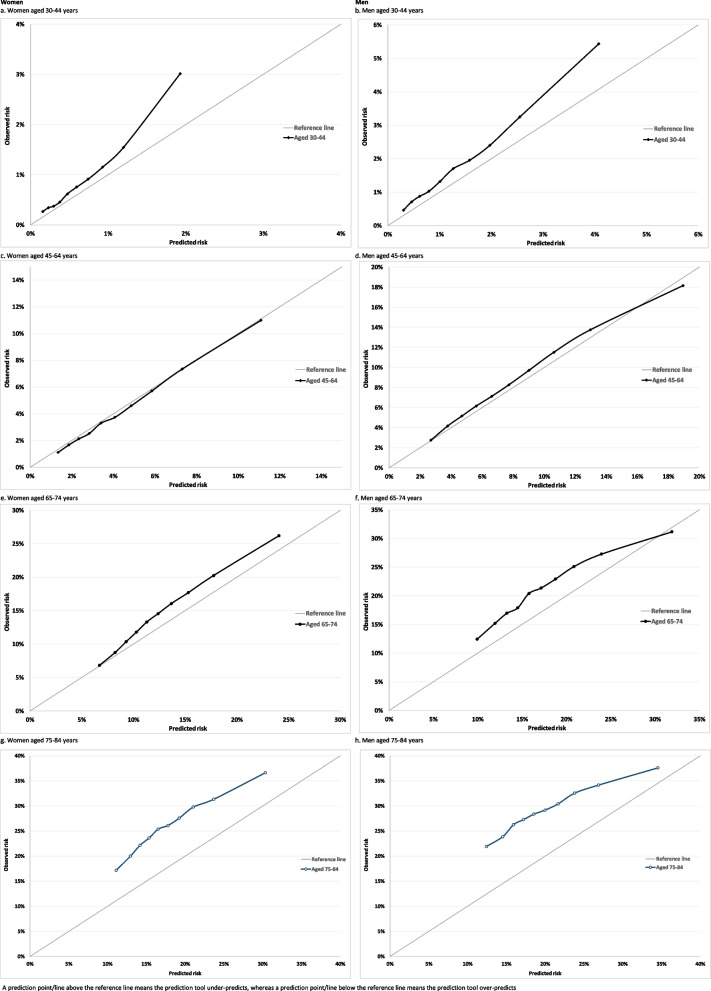
Calibration in women (left hand) and men (right hand) stratified by age

**Fig. 3:**
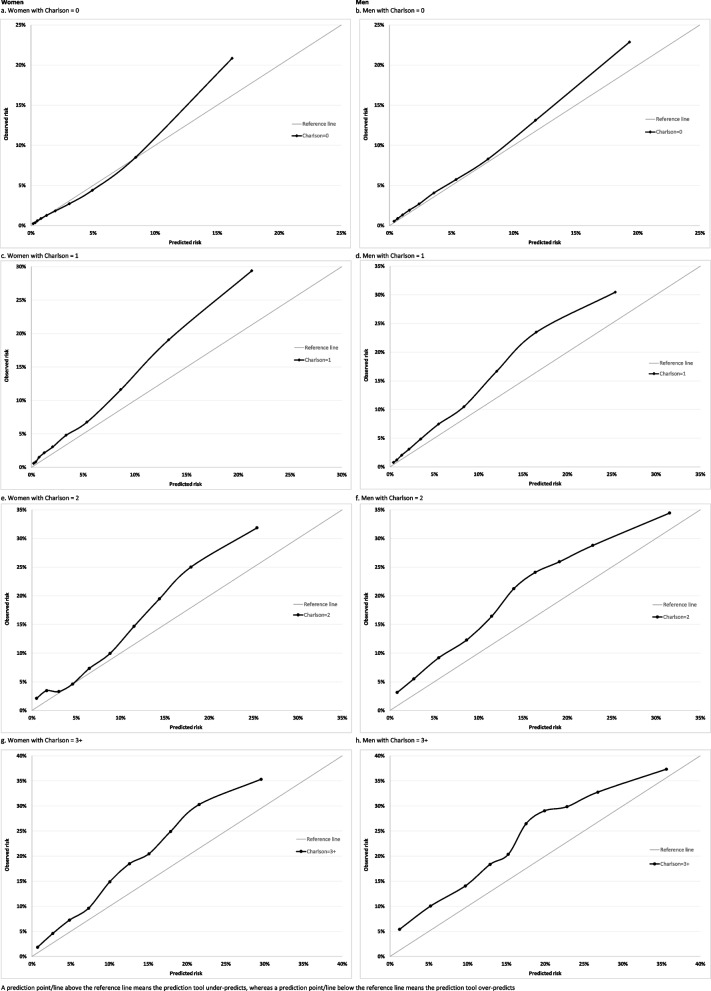
Calibration in women (left hand) and men (right hand) stratified by Charlson Comorbidity Index

In the whole population over 10-years, there was reasonable calibration (with some under-prediction) in the eight deciles of lowest predicted risk with QRiskLifetime, but considerable under-prediction in the two deciles of highest predicted risk (Fig. 1). Stratified by age (Fig. 2), calibration was good in people aged 45–64, with under-prediction in all other age-groups which was largest in people aged 75–84. Stratified by CCI, there was under-prediction at all levels of morbidity which was more marked at higher levels of predicted risk and at higher levels of multimorbidity (Fig. 3).

In the reclassification analysis (Tables 3, 5 and 4), compared to QRISK3, QRiskLifetime classified fewer people as having 10-year risk ≥ 10%. QRISK3 classified 239,396 (19.0%) women as high-risk, compared to 194,411 (15.4%) women classified as high-risk by QRiskLifetime over 10-years. QRISK3 classified 341,962 (28.0%) men as high-risk, compared to 276,369 (22.6%) men classified as high-risk by QRiskLifetime over 10-years (Table 3). 15.1% of women were classified as high-risk (≥ 10% over 10-years) by both tools, 3.9% as only high-risk by QRISK3 and 0.3% as only high-risk by QRiskLifetime (with the remaining 80.7% <10% on both scores). 21.9% of men were classified as high-risk (≥ 10% over 10-years) by both tools, 6.1% as only high-risk by QRISK3 and 0.7% as only high-risk by QRiskLifetime.

**Table 3:** Reclassification as high or low risk by QRiskLifetime compared to QRISK3 with both predicting risk over 10-years

	QRiskLifetime < 10% at 10 years No. (%) of all women or all men	QRiskLifetime ≥ 10% at 10 years No. (%) of all women or all men	Total recommended for treatment by each tool
**Women**			
QRISK3 < 10%	1,017,314 (80.7)	3619 (0.3)	QRiskLifetime recommends 15.4% for treatment
QRISK3 ≥ 10%	48,604 (3.9)	190,792 (15.1)	QRISK3 recommends 19.0% for treatment
**Men**			
QRISK3 < 10%	872,474 (71.3)	8829 (0.7)	QRiskLifetime recommends 22.6% for treatment
QRISK3 ≥ 10%	74,422 (6.1)	267,540 (21.9)	QRisk3 recommends 28.0% for treatment

Cohen’s Kappa: Women 0.86 (95% CI 0.85 to 0.86), men 0.82 (95% CI 0.82 to 0.82)

**Table 4:** Characteristics of people recommended for treatment

	Number (%) recommended for treatment	No. (%) with a CVD event	Number Needed to Treat (NNT)^b^	Mean (SD) age	Mean (SD) TC:HDL ratio	Mean (SD) SBP (mmHg)	Mean (SD) BMI (kg/m^2^)	Treated HT % (95%CI)	Current smoker % (95%CI)	Family history premature CVD % (95% CI)	Minority ethnic background % (95% CI)
**Women**											
QRISK3 predicted risk at 10 years ≥ 10%	239,396 (19.0)	28,373 (11.9)	34	71.3 (8.2)	3.8 (0.8)	143.9 (17.0)	26.8 (4.5)	31.9 (31.7–32.1)	18.1 (17.9–18.2)	6.3 (6.2–6.4)	3.0 (2.9–3.1)
QRiskLifetime predicted risk at 10 years ≥ 10%	194,411 (15.4)	25,641 (13.2)	30	73.3 (7.0)	3.8 (0.8)	145.0 (17.0)	26.8 (4.4)	36.2 (36.0-36.4)	15.9 (15.7–16.0)	7.8 (7.7–7.9)	3.2 (3.1–3.2)
QRiskLifetime predicted lifetime risk ≥ 32.9% ^a^	239,396 (19.0)	9652 (4.0)	99	50.5 (12.6)	4.0 (1.1)	134.9 (20.0)	28.9 (5.6)	29.4 (29.2–29.6)	21.3 (21.2–21.5)	36.3 (36.2–36.5)	20.8 (20.6–20.9)
**Men**											
QRISK3 predicted risk at 10 years ≥ 10%	341,962 (28.0)	37,026 (10.8)	37	63.8 (9.6)	4.3 (0.9)	140.2 (15.5)	27.1 (3.7)	19.6 (19.5–19.8)	26.1 (26.0-26.2)	7.2 (7.1–7.2)	3.2 (3.1–3.2)
QRiskLifetime predicted risk at 10 years ≥ 10%	276,369 (22.6)	33,450 (12.1)	33	66.2 (8.5)	4.3 (0.9)	140.8 (15.6)	27.1 (3.7)	22.3 (22.2–22.5)	23.4 (23.2–23.6)	8.3 (8.2–8.4)	3.3 (3.3–3.4)
QRiskLifetime predicted lifetime risk ≥ 39.6% ^a^	341,962 (28.0)	14,725 (4.3)	100	46.3 (10.4)	4.9 (0.9)	135.7 (15.2)	29.1 (4.1)	15.0 (14.9–15.2)	26.4 (26.2–26.5)	20.0 (19.9–20.2)	13.1 (13.0-13.2)

*TC HDL ratio * Total cholesterol, HDLCholesterol ration, *SBP *Systolic blood pressure, *HT* Hypertension

^a^There is no recommended threshold of lifetime risk above which treatment is recommended, so for comparison purposes, QRiskLifetime thresholds are defined to include the same number of patients recommended for treatment as QRISK3 10-year risk ≥ 10% (i.e. the 19.0% of women and 28.0% of men at highest lifetime risk are ‘recommended’ for treatment to match the 19.0% of women and 28.0% of men with QRISK3 10-year risk ≥ 10%)

^b^Assuming a 25% risk reduction with primary prevention using statins with treatment taken by all people recommended for treatment

In women, compared to QRISK3, QRISKLifetime slightly improved classification in those who did not experience an event (Net Reclassification Index NRI- = 0.035, 95% CI 0.034 to 0.035), but worsened classification in those who did experience an event (NRI+ = -0.080, 95% CI -0.082 to -0.077), with overall NRI − 0.045 (95% confidence interval − 0.047 to -0.042; in other words, overall 4.5% of participants are incorrectly reclassified). In men, compared to QRISK3, QRISKLifetime slightly improved classification in those who did not experience an event (NRI- = 0.054, 95% CI 0.054 to 0.054), but worsened classification in those who did experience an event (NRI+ = -0.083, 95% CI -0.084 to -0.082), with overall NRI − 0.029 (95% confidence interval − 0.030 to -0.028).

Those recommended for treatment by QRiskLifetime based on 10-year risk were slightly older than those recommended by QRISK3, but patient characteristics were otherwise similar (Table 4). Fewer people were recommended for treatment by QRiskLifetime based on 10-year risk but the percentage experiencing an event was higher (estimated number needed to treat (NNT) from statin prescription to prevent one event in women 34 for QRISK3 vs. 30 for QRiskLifetime; for men 37 vs. 33).

By design, thresholds of predicted lifetime risk for “recommending treatment” were chosen so that exactly the same number of people at highest lifetime risk were identified as were identified by QRISK3 10-year risk ≥ 10% (Table 5). Both tools therefore “recommended” 19.0% of women and 28.0% of men for treatment. Only 5.3% of all women were identified as high-risk by both tools, with a different 13.7% identified as high-risk by one or other of the prediction tools. Similarly, 8.9% of men were identified as high-risk by both prediction tools and a different 19.1% by one or other of the tools. Compared to people identified as high-risk by QRISK3, those with highest predicted lifetime risk were much younger, had lower mean systolic blood pressure, and a lower proportion with treated hypertension, but much higher proportions with family history of premature CVD and from a minority ethnic background, and somewhat higher mean total cholesterol:HDL cholesterol ratio and BMI (Table 4). Compared to those recommended for treatment based on 10-year predicted risk, there were fewer CVD events observed in people at the highest predicted lifetime risk, and the estimated NNT to prevent one CVD event from statin treatment was 99 in women and 100 in men.

**Table 5:** Reclassification as high or low risk by QRiskLifetime predicting lifetime risk compared to QRISK3 predicting risk over 10-years

	QRiskLifetime < 32.9% (women) or < 39.6% (men)^a^ No. (%) of all women or all men	QRiskLifetime ≥ 32.9% (women) or ≥ 39.6% (men)^a^ No. (%) of all women or all men	Total recommended for treatment by each tool^a^
**Women**			
QRISK3 < 10%	847,786 (67.3)	173,147 (13.7)	QRiskLifetime recommends 19.0% for treatment
QRISK3 ≥ 10%	173,147 (13.7)	66,249 (5.3)	QRISK3 recommends 19.0% for treatment
**Men**			
QRISK3 < 10%	647,949 (53.0)	233,354 (19.1)	QRiskLifetime recommends 28.0% for treatment
QRISK3 ≥ 10%	233,354 (19.1)	108,608 (8.9)	QRisk3 recommends 28.0% for treatment

^a^There is no recommended threshold of lifetime risk above which treatment is recommended, so for comparison purposes, QRiskLifetime thresholds are defined to identify exactly the same number of patients as those identified by QRISK3 as having 10-year risk ≥ 10% (i.e. the 19.0% of women and 28.0% of men at highest lifetime risk are ‘recommended’ for treatment to match the 19.0% of women and 28.0% of men with QRISK3 10-year risk ≥ 10%)

## Discussion

Similar to the internal validation study [3], this independent evaluation of the QRiskLifetime CVD risk prediction tool finds that it has excellent discrimination in the whole population over a 10-year prediction horizon, but discrimination is poor to moderate in age and CCI subgroups. In terms of calibration over a 10-year prediction horizon, there was some under-prediction in the whole population. Stratified by age, calibration was excellent in women aged 45–64 and good in men aged 45–64, but there was considerable under-prediction in other age-groups which was larger in younger people at higher risk and in all older people.

Over a 10-year prediction horizon at the 10% risk threshold recommended by NICE [9], QRiskLifetime recommended fewer people for statin treatment (15.4% of women and 22.6% of men) than QRISK3 (19.0% of women and 28.0% of men), although the estimated NNT to prevent one CVD event over 10-years was slightly lower for QRiskLifetime.

Comparing those recommended for treatment by QRISK3 predicted 10-year risk ≥ 10% versus the same proportion at highest estimated lifetime risk by QRiskLifetime, the populations recommended for treatment were markedly different, with those at highest predicted lifetime risk being considerably younger, being much more likely to have a family history of premature CVD and be from a minority ethnic background. Treating the same number of patients at highest predicted lifetime risk as the number with QRISK3 10-year risk ≥ 10%, the estimated NNT with a statin to prevent one CVD event over 10 years was approximately three times higher compared to QRISK3 (in women 99 vs. 34; in men 100 vs. 37). Any benefit of treating those at the highest lifetime rather than the highest 10-year CVD risk is therefore considerably deferred.

Important strengths of the study are the use of population data and study design, conduct and reporting consistent with methodology recommendations [13, 24], publishing all codesets used [6], accounting for competing mortality risks, and examining performance in key subgroups. Key limitations are those common to studies using linked routine data. In the context of lifetime risk prediction, the most important of these is the relatively short follow-up of study participants although this is similar to other studies in this context. Constraining validation to events observed over ten years therefore does not allow evaluation of the potential benefit of longer-term prediction in younger people. However, even if data were available, then evaluating model performance over 20 or more years may reduce applicability to contemporary risk prediction given declining secular trends in age-standardised incident CVD. A further limitation is the high proportion of people with missing data. As with the derivation study and other studies, we used multiple imputation but the assumption that data is missing at random may be incorrect [6, 25].

Brotons et al. also found substantial differences in who was recommended for treatment by 10-year vs. lifetime risk prediction tools, but did not validate lifetime predictions. [4] Like QRiskLifetime, the LIFE-CVD risk prediction tool estimates both 10-year and lifetime CVD risk. LIFE-CVD derivation was in a US dataset, with validation in several European cohorts, with reasonable discrimination and whole population calibration at 10-years follow-up [5]. However, unlike this study, calibration was not examined stratified by age and if calibration is less good in older people, then the implication would be that lifetime estimates are also not well calibrated.

Guidelines currently only recommend lifetime CVD risk prediction as an adjunct to 10-year risk prediction [1], but without specifying any risk thresholds for action. In the absence of lifetime follow-up data and in the context of falling age-standardised rates of incidence CVD, there is no way to directly evaluate how well lifetime estimates perform, but given the observed under-prediction over 10-years in every age-group in this study, we believe that QRiskLifetime is likely to under-predict risk over a lifetime. It is unclear whether similar issues apply to other lifetime risk tools because calibration has not been examined in subgroups of age [5, 26]. More broadly, for all CVD risk prediction, excellent discrimination and calibration in the whole population does not mean that discrimination and calibration are good enough in important subgroups [27], and validation should explore subgroup performance [6].

Even if a lifetime prediction tool were well calibrated in different age-groups, lifetime risk prediction requires an assumption that future risk in younger people will be the same as the risk observed in older people now. Given large falls in CVD incidence in recent decades and continuing change in CVD risk profiles (declining smoking but increasing obesity and diabetes), this assumption is a very strong one. Furthermore, although lifetime expected benefit is greater if treatment is started at a younger age, this study finds that the expected benefit in the medium-term (over 10-years) is considerably smaller. Given the lack of direct evidence, early treatment based on predicted lifetime risk therefore requires a leap of faith by both patient and clinician that additional years of early treatment will lead to larger benefit in the distant future. In that context, careful explanation of predicted risks is needed, and patient preferences are critical to take into account [5, 28].

A key limitation in the field is that UK and other linked routine data resources used to derive and validate CVD risk prediction usually suffer from limited follow-up because patients are lost when they deregister with a participating practice or organisation. We constrained validation of performance to 10-years to allow a direct comparison with QRISK3, but even without this, follow-up is constrained by deregistration from participating practices, and very long follow-up also requires the use of very historical baseline data when data quality is poorer and CVD incidence was higher. Improvements in data linkage and increasing access to whole population data have the potential to significantly improve observability over long period of follow-up, and deriving and validating new prediction tools in these datasets which account for competing mortality risk is a priority.

More broadly, lifetime CVD risk prediction is an attempt to deal with a key problem of 10-year CVD risk prediction: that younger people at high risk of premature CVD often do not have 10-year CVD risk that exceeds current threshold for treatment. Using age-stratified 10-year risk thresholds might mitigate this problem [28], but risks large proportions of people being recommended for lifelong medication that most will not benefit from. With advances in cardiac imaging, alternative strategies include using coronary artery calcium scoring [28] or CT coronary angiography (CTCA) to screen people at increased predicted risk for asymptomatic coronary artery disease, and to treat the diseased rather than the at-risk. Early diagnosis and treatment is an attractive strategy given the problems of risk prediction over long periods of time, but while such a strategy using CTCA has been shown to be effective in people with chest pain [29], its value in a true primary prevention population is uncertain and needs to be established [30].

## Conclusion

QRiskLifetime under-predicts risk over a 10-year prediction horizon in all patients except women aged 45–64, and is therefore likely to under-predict risk over a lifetime. Given limited follow-up in derivation and validation studies, any lifetime prediction in younger people requires the strong assumption that age-stratified incidence of CVD will remain stable over decades. Compared to treatment based on 10-year risk, treatment based on lifetime risk therefore requires a considerably larger leap of faith on the part of clinicians and patients.

## Supplementary Information

**Additional file 1: Supplementary Table 1**. Missing data. **Supplementary Table S2**. Incidence of cardiovascular disease during entire period of follow-up. **Supplementary Table S3**. Incidence of non-cardiovascular death during entire period of follow-up. **Figure S1**. Incidence of cardiovascular disease and non-cardiovascular death in men and women. 

## Data Availability

The data that support the findings of this study are available from Clinical Practice Research Datalink (https://cprd.com/), but restrictions apply to the availability of these data, which were used under license for the current study, and so are not publicly available. Codelists defining all variables used in analysis are published as supplementary material to 10.1016/S2666-7568(21)00088-X. The data controller is the Clinical Practice Research Datalink (CPRD), and under the data licence granted, the authors are not allowed to share data. Researchers can apply to CPRD directly for access to the raw data (https://cprd.com/).
