# Supplementary material for: External validation of the QLifetime cardiovascular risk prediction tool: population cohort study
Source: BMC Cardiovasc Disord. 2023 Apr 15;23:194. doi: 10.1186/s12872-023-03209-8 (PMC10105395; doi:10.1186/s12872-023-03209-8)
Supplement: Supplementary file 1 — Additional file 1: Supplementary Table 1. Missing data. Supplementary Table S2. Incidence of cardiovascular disease during entire period of follow-up. Supplementary Table S3. Incidence of non-cardiovascular death during entire period of follow-up. Figure S1. Incidence of cardiovascular disease and non-cardiovascular death in men and women. [file 12872_2023_3209_MOESM1_ESM.docx]

**External validation of the QLifetime cardiovascular risk prediction tool: population cohort study: Supplementary File**

Corresponding author:

Bruce Guthrie, Doorway 3, Old Medical School, University of Edinburgh, Teviot Place, Edinburgh EH8 9AG

[bruce.guthrie@ed.ac.uk](mailto:bruce.guthrie@ed.ac.uk)

[Supplementary table 1: Missing data 2](#_Toc101606649)

[Supplementary table S2: Incidence of cardiovascular disease during entire period of follow-up 3](#_Toc101606650)

[Supplementary table S3: Incidence of non-cardiovascular death during entire period of follow-up 4](#_Toc101606651)

[Figure S1: Incidence of cardiovascular disease and non-cardiovascular death in men and women 5](#_Toc101606652)

#### Supplementary table 1: Missing data

|  | How missingness  was handled  in analysis | Women external validation cohort   N=1260329  No (%) missing data | Men external  validation cohort  N=1223265 No (%) missing data | QRisk Lifetime internal validation cohort  N=1267159 |
| --- | --- | --- | --- | --- |
| Age  Sex  Socioeconomic status | Never missing  Never missing  Excluded from cohort | 0  0  0 | 0  0  0 | 0  0  0 |
| Body mass index (BMI) | Imputed | 365002 (29.0) | 510029 (41.7) | 317725 (25.1) |
| Total cholesterol:HDL cholesterol (TC:HDL) ratio | Imputed | 1046723 (83.1) | 1019032 (83.3) | 912036 (72.0) |
| Systolic blood pressure | Imputed | 209942 (16.7) | 401962 (32.9) | 185215 (14.6) |
| Smoking status | Imputed | 273978 (21.7) | 388161 (31.7) | 145 690 (11.5) |
| Ethnicity | Assumed to be white | 263272 (20.9) | 416024 (34.0) | Not available |
| Condition variables | Assumed to be absent if no record | NA | NA | NA |

#### Supplementary table S2: Incidence of cardiovascular disease during entire period of follow-up

|  | Women |  |  | Men |  |  |
| --- | --- | --- | --- | --- | --- | --- |
| Agegroup | Incident CVD  No. of patients | Total Follow-up  Years | Rate (95% CI) per 1000 person years | Incident CVD  No. of patients | Total Follow-up  Years | Rate (95% CI) per 1000 person years |
| 30-34 | 560 | 1153862 | 0.5 (0.4,0.5) | 974 | 1144138 | 0.9 (0.8,0.9) |
| 35-39 | 1,098 | 1238221 | 0.9 (0.8,0.9) | 2,185 | 1249707 | 1.7 (1.7,1.8) |
| 40-44 | 1,762 | 1136909 | 1.5 (1.5,1.6) | 3,519 | 1145201 | 3.1 (3.0,3.2) |
| 45-49 | 2,221 | 939971 | 2.4 (2.3,2.5) | 4,779 | 916468 | 5.2 (5.1,5.4) |
| 50-54 | 2,739 | 812590 | 3.4 (3.2,3.5) | 5,628 | 749133 | 7.5 (7.3,7.7) |
| 55-59 | 3,776 | 754370 | 5.0 (4.8,5.2) | 6,877 | 654761 | 10.5 (10.3,10.8) |
| 60-64 | 4,053 | 509885 | 7.9 (7.7,8.2) | 6,249 | 409070 | 15.3 (14.9,15.7) |
| 65-69 | 4,864 | 387189 | 12.6 (12.2,12.9) | 6,126 | 289067 | 21.2 (20.7,21.7) |
| 70-74 | 6,169 | 304427 | 20.3 (19.8,20.8) | 6,157 | 207295 | 29.7 (29.0,30.4) |
| 75-80 | 7,117 | 237437 | 30.0 (29.3,30.7) | 5,758 | 142794 | 40.3 (39.3,41.4) |
| 80-84 | 7,835 | 177496 | 44.1 (43.2,45.1) | 4,501 | 85637 | 52.6 (51.1,54.1) |
| Total | 42,194 | 7652358 | 5.5 (5.5,5.6) | 52,753 | 6993272 | 7.5 (7.5,7.6) |

#### Supplementary table S3: Incidence of non-cardiovascular death during entire period of follow-up

|  | Women |  |  | Men |  |  |
| --- | --- | --- | --- | --- | --- | --- |
| Agegroup | Incident CVD  No. of patients | Total Follow-up  Years | Rate (95% CI) per 1000 person years | Incident CVD  No. of patients | Total Follow-up  Years | Rate (95% CI) per 1000 person years |
| 30-34 | 643 | 1153862 | 0.6 (0.5,0.6) | 916 | 1144138 | 0.8 (0.8,0.9) |
| 35-39 | 1,080 | 1238221 | 0.9 (0.8,0.9) | 1,394 | 1249707 | 1.1 (1.1,1.2) |
| 40-44 | 1,556 | 1136909 | 1.4 (1.3,1.4) | 1,752 | 1145201 | 1.5 (1.5,1.6) |
| 45-49 | 2,106 | 939971 | 2.2 (2.1,2.3) | 2,287 | 916468 | 2.5 (2.4,2.6) |
| 50-54 | 2,704 | 812590 | 3.3 (3.2,3.5) | 2,883 | 749133 | 3.8 (3.7,4.0) |
| 55-59 | 3,624 | 754370 | 4.8 (4.7,5.0) | 4,161 | 654761 | 6.4 (6.2,6.6) |
| 60-64 | 3,820 | 509885 | 7.5 (7.3,7.7) | 4,052 | 409070 | 9.9 (9.6,10.2) |
| 65-69 | 4,526 | 387189 | 11.7 (11.4,12.0) | 4,753 | 289067 | 16.4 (16.0,16.9) |
| 70-74 | 5,894 | 304427 | 19.4 (18.9,19.9) | 5,677 | 207295 | 27.4 (26.7,28.1) |
| 75-80 | 8,039 | 237437 | 33.9 (33.1,34.6) | 6,562 | 142794 | 46.0 (44.9,47.1) |
| 80-84 | 10,263 | 177496 | 57.8 (56.7,58.9) | 6,405 | 85637 | 74.8 (73.0,76.6) |
| Total | 44,255 | 7652358 | 5.8 (5.7,5.8) | 40,842 | 6993272 | 5.8 (5.8,5.9) |

#### Figure S1: Incidence of cardiovascular disease and non-cardiovascular death in men and women

| **Women**  **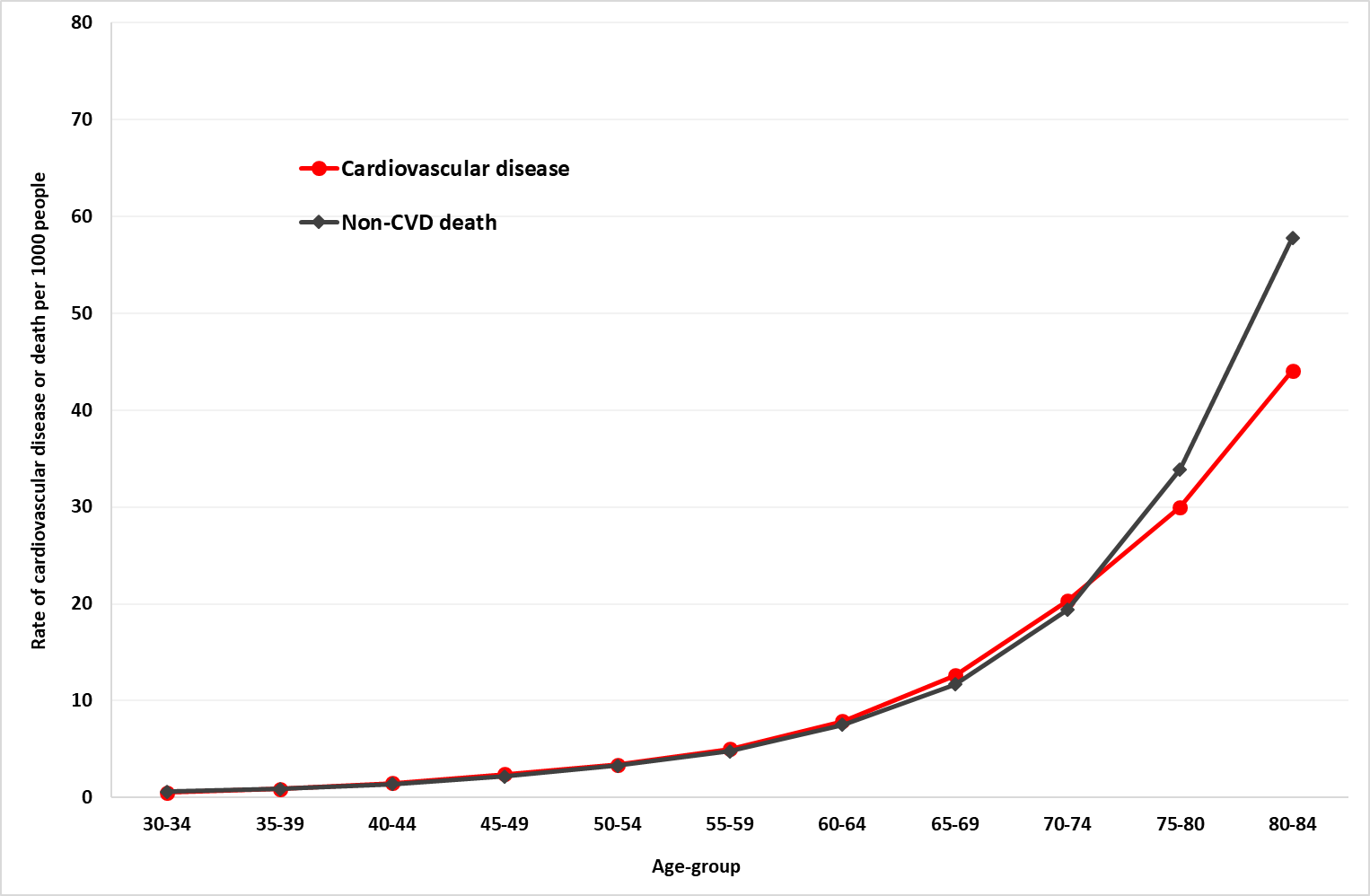** |
| --- |
| **Men**  **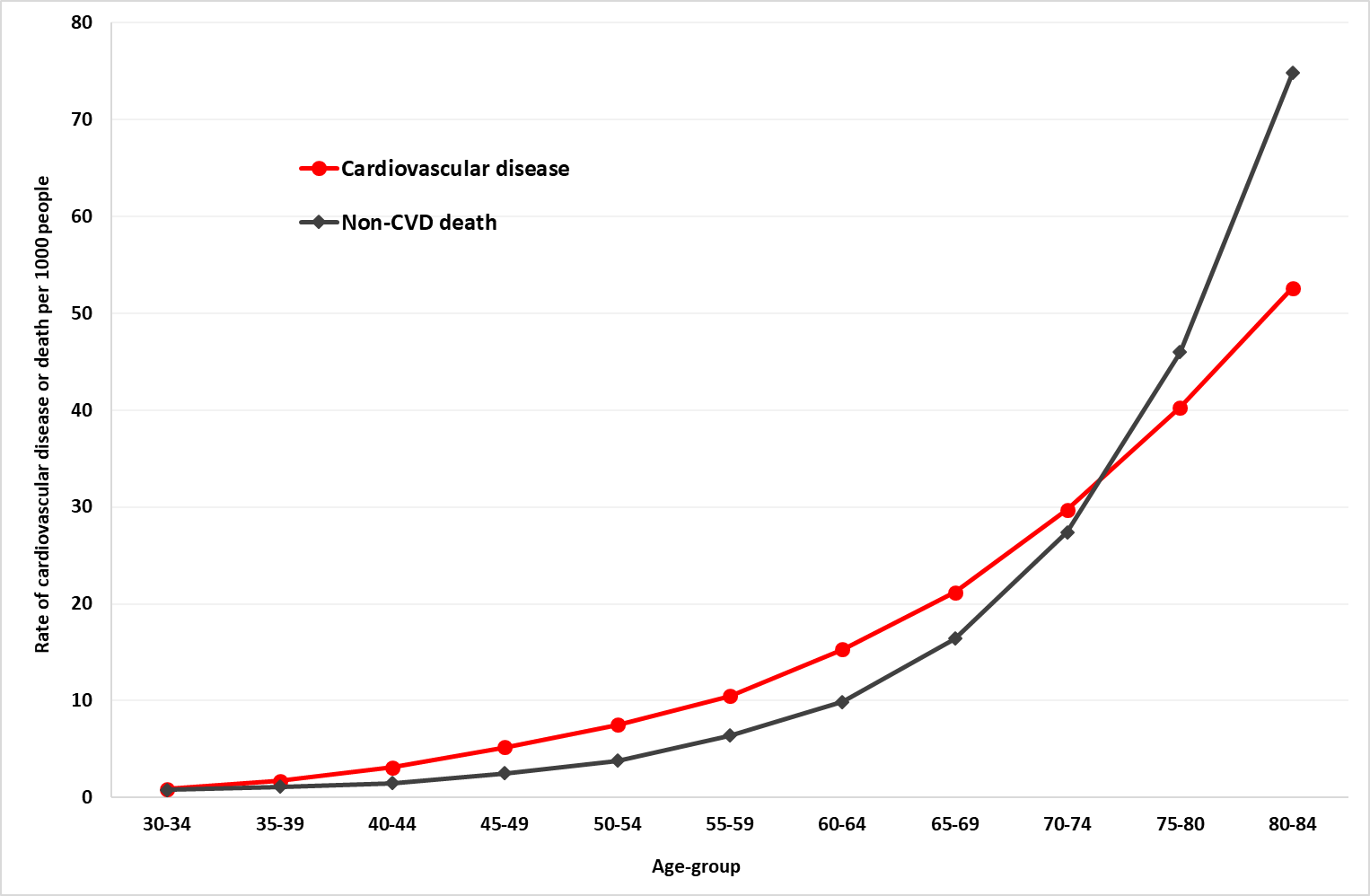** |
